# Antisense Therapy Attenuates Phospholamban p.(Arg14del) Cardiomyopathy in Mice and Reverses Protein Aggregation

**DOI:** 10.3390/ijms23052427

**Published:** 2022-02-22

**Authors:** Tim R. Eijgenraam, Nienke M. Stege, Vivian Oliveira Nunes Teixeira, Remco de Brouwer, Elisabeth M. Schouten, Niels Grote Beverborg, Liu Sun, Daniela Später, Ralph Knöll, Kenny M. Hansson, Carl Amilon, David Janzén, Steve T. Yeh, Adam E. Mullick, Peter van der Meer, Rudolf A. de Boer, Herman H. W. Silljé

**Affiliations:** 1 University Medical Center Groningen, Department of Cardiology, University of Groningen, Antonius Deusinglaan 1, 9713 AV Groningen, The Netherlands; t.r.eijgenraam@umcg.nl (T.R.E.); n.m.stege@umcg.nl (N.M.S.); v.oliveira.nunes.teixeira@umcg.nl (V.O.N.T.); r.de.brouwer@umcg.nl (R.d.B.); e.m.schouten@umcg.nl (E.M.S.); n.grote.beverborg@umcg.nl (N.G.B.); l.sun@umcg.nl (L.S.); p.van.der.meer@umcg.nl (P.v.d.M.); r.a.de.boer@umcg.nl (R.A.d.B.); 2 Netherlands Heart Institute, Moreelsepark 1, 3511 EP Utrecht, The Netherlands; 3 Bioscience Cardiovascular, Research and Early Development, Cardiovascular, Renal and Metabolism (CVRM), BioPharmaceuticals R&D, AstraZeneca, Pepparedsleden 1, 431 50 Gothenburg, Sweden; daniela.spaeter@astrazeneca.com (D.S.); ralph.knoell@astrazeneca.com (R.K.); kenny.m.hansson@astrazeneca.com (K.M.H.); 4 Projects, Research and Early Development, Cardiovascular, Renal and Metabolism, BioPharmaceuticals R&D, AstraZeneca, Pepparedsleden 1, 431 50 Gothenburg, Sweden; carl.amilon@telia.com; 5 Drug Metabolism and Pharmacokinetics, Research and Early Development, Cardiovascular, Renal and Metabolism, BioPharmaceuticals R&D, AstraZeneca, Pepparedsleden 1, 431 50 Gothenburg, Sweden; david.janzen@astrazeneca.com; 6 Ionis Pharmaceuticals, 2855 Gazelle Court, Carlsbad, CA 92010, USA; syeh@ionisph.com (S.T.Y.); amullick@ionisph.com (A.E.M.)

**Keywords:** phospholamban, genetic mutation, protein aggregation, cardiomyopathy, heart failure, gene therapy

## Abstract

Inherited cardiomyopathy caused by the p.(Arg14del) pathogenic variant of the phospholamban (*PLN*) gene is characterized by intracardiomyocyte PLN aggregation and can lead to severe dilated cardiomyopathy. We recently reported that pre-emptive depletion of PLN attenuated heart failure (HF) in several cardiomyopathy models. Here, we investigated if administration of a *Pln*-targeting antisense oligonucleotide (ASO) could halt or reverse disease progression in mice with advanced PLN-R14del cardiomyopathy. To this aim, homozygous PLN-R14del (PLN-R14 ^Δ/Δ^) mice received PLN-ASO injections starting at 5 or 6 weeks of age, in the presence of moderate or severe HF, respectively. Mice were monitored for another 4 months with echocardiographic analyses at several timepoints, after which cardiac tissues were examined for pathological remodeling. We found that vehicle-treated PLN-R14 ^Δ/Δ^ mice continued to develop severe HF, and reached a humane endpoint at 8.1 ± 0.5 weeks of age. Both early and late PLN-ASO administration halted further cardiac remodeling and dysfunction shortly after treatment start, resulting in a life span extension to at least 22 weeks of age. Earlier treatment initiation halted disease development sooner, resulting in better heart function and less remodeling at the study endpoint. PLN-ASO treatment almost completely eliminated PLN aggregates, and normalized levels of autophagic proteins. In conclusion, these findings indicate that PLN-ASO therapy may have beneficial outcomes in PLN-R14del cardiomyopathy when administered after disease onset. Although existing tissue damage was not reversed, further cardiomyopathy progression was stopped, and PLN aggregates were resolved.

## 1. Introduction

Dilated cardiomyopathy (DCM) is the second most common cause of heart failure (HF) with reduced ejection fraction (HFrEF) after coronary artery disease [1]. It has been estimated that up to 40% of DCM cases have a genetic cause [1]. Most frequently, these variants are identified in cardiac genes encoding proteins of the sarcomere, ion channels or nuclear membrane [1]. The p.(Arg14del) pathogenic variant of the phospholamban (*PLN*) gene (PLN-R14del) is a Dutch founder mutation with a high prevalence in DCM and arrhythmogenic cardiomyopathy (ACM) patients [2]. PLN is a cardiac protein that regulates the uptake of cytosolic calcium ions into the sarcoplasmic reticulum (SR) of cardiomyocytes via inhibition of sarco/endoplasmic reticulum Ca^2+^-ATPase (SERCA) activity [3]. Upon stimulation of the β-adrenergic signaling pathway, protein kinase A (PKA) phosphorylates PLN proteins to relieve SERCA inhibition, resulting in increased lusitropy and inotropy [3]. Although the regulatory effect of PLN on SERCA has attracted most attention, contradictory results have been published on the effect of PLN-R14del on the PLN-SERCA interaction [4,5], and recent data also indicate nuclear [6,7] and mitochondrial [8,9] functions of PLN, thus the exact disease mechanisms of pathogenic variants remain elusive. Interestingly, the PLN-R14del pathogenic variant is characterized by dense perinuclear protein aggregation [10], a phenomenon shared with several other pathogenic DCM and ACM gene variants [11,12,13]. Since protein aggregation is a well-known pathogenic hallmark of many diseases and in aging [14], PLN protein aggregation has been implicated as a potential mechanism driving cardiac remodeling in PLN-R14del cardiomyopathy [10,15]. We have recently developed a mouse model carrying the PLN-R14del pathogenic variant, which recapitulates most disease characteristics that are observed in human patients [16]. Mice that are homozygous for the PLN-R14del pathogenic variant (PLN-R14 ^Δ/Δ^) develop cardiomyopathy, PLN protein aggregation and HF in an accelerated manner with early mortality within 2 months, providing an opportunity to screen novel therapies [15,16].

Antisense therapy uses antisense oligonucleotides (ASOs) to directly alter mRNA processing or degradation without the need for genetic modifications [17]. Several ASOs have been approved for clinical use, e.g., targeting dystrophin (*DMD*) in Duchenne muscular dystrophy, transthyretin (*TTR*) in familial amyloid polyneuropathy (FAP) or survival of motor neuron 2 (*SMN2*) in spinal muscular atrophy (SMA), and dozens of ASOs are currently being investigated in clinical trials [17]. Since PLN-R14del proteins resulting from the p.(Arg14del) pathogenic variant are the root cause of PLN-R14del cardiomyopathy, elimination of the mutant protein could be a promising therapeutic approach. We have recently demonstrated that pre-emptive reduction in *Pln* mRNA using a PLN-ASO could delay onset and reduce severity of HF in multiple experimental models [18]. However, patients are often identified after disease onset, upon presentation of HF-related symptoms. Additionally, as there is great clinical heterogeneity amongst mutation carriers that are found before disease onset (e.g., through family screening), risk prediction is challenging, and in clinical practice, therapy is usually initiated after disease onset [19]. Thus, the effect of PLN inhibition in a clinically relevant setting of established cardiomyopathy remains yet unexplored. In this study, we aimed to investigate whether administration of a PLN-ASO could resolve pre-existing protein aggregates, and halt HF progression in PLN-R14 ^Δ/Δ^ mice with established cardiac remodeling and left-ventricular (LV) dysfunction.

## 2. Results

### 2.1. PLN-ASO Treatment Prolongs Survival of PLN-R14 ^Δ/Δ^ Mice

**Figure 1:**
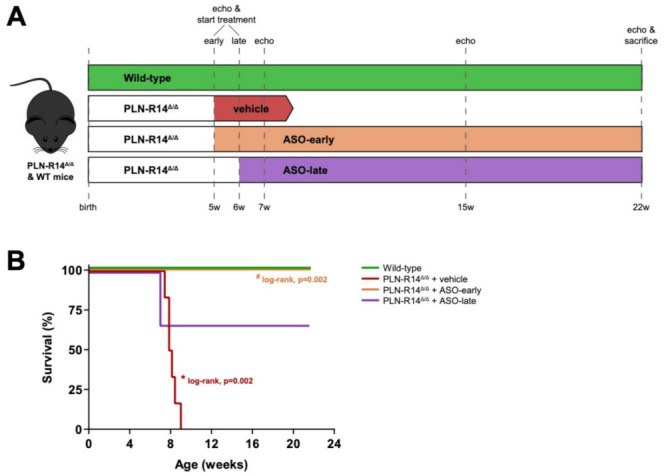
Schematic overview of the study design and survival curve. (**A**) PLN-R14 ^Δ/Δ^ mice were randomized to administration of PLN-ASO or vehicle (saline). PLN-ASO treatment was initiated at 5 or 6 weeks of age (ASO-early and ASO-late, respectively), when PLN-R14 ^Δ/Δ^ mice had developed moderate or severe heart failure, respectively. Heart function was determined using echocardiography at treatment start and at the age of 7, 15 and 22 weeks. Survival of PLN-R14 ^Δ/Δ^ mice was monitored up to 22 weeks of age, when all mice were sacrificed for histological and molecular tissue analyses. (**B**) Percentage survival of WT mice and PLN-R14 ^Δ/Δ^ mice receiving early or late PLN-ASO treatment or vehicle injections to a maximum of 22 weeks of age (*n* = 6 per group). * *p* < 0.05 vs. WT, ^#^ *p* < 0.05 vs. PLN-R14 ^Δ/Δ^ + vehicle (log-rank test).

To investigate whether administration of a PLN-ASO could halt or even reverse established PLN-R14del cardiomyopathy, we aimed to initiate treatment when PLN-R14 ^Δ/Δ^ mice had considerable HF. Based on previously published data on the development of DCM in PLN-R14 ^Δ/Δ^ mice [15,16], we decided to start PLN-ASO injections at 5 (ASO-early) or 6 (ASO-late) weeks of age when mice were shown to exhibit moderate (27.5 ± 0.2% fractional shortening (FS) vs. 36.2 ± 1.1% in age-matched wild-type (WT) controls) or severe (13.6 ± 2.3% FS) LV dysfunction with ventricular dilatation, respectively (Figure 1A and Figure S1). As expected [16], vehicle-treated PLN-R14 ^Δ/Δ^ mice reached a humane endpoint at 8.1 ± 0.5 weeks of age (Figure 1B). Both early and late PLN-ASO treatment extended the survival of PLN-R14 ^Δ/Δ^ mice for at least 3 months until sacrifice, except for two mice of the late PLN-ASO administration group that experienced cardiac arrest upon induction of anesthesia for the echocardiogram at the age of 7 weeks.

### 2.2. Heart Failure Progression Is Halted by PLN-ASO Treatment

**Figure 2:**
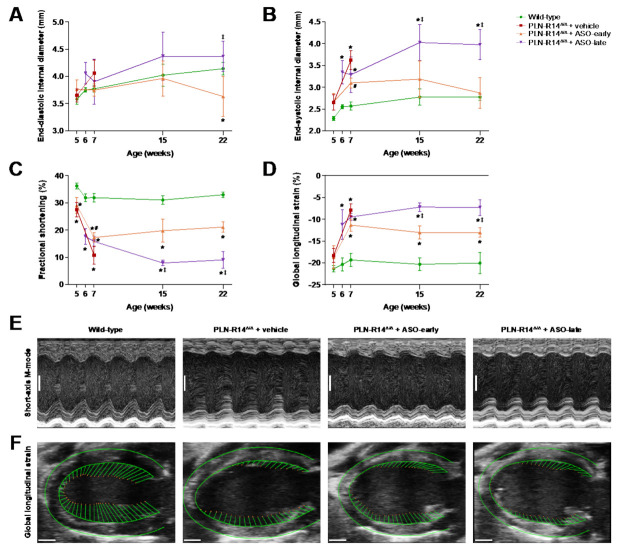
Heart failure progression was halted by PLN-ASO treatment. (**A**-**D**) Left-ventricular end-diastolic (**A**) and end-systolic (**B**) internal diameters, percentage fractional shortening (**C**) and percentage global longitudinal strain (**D**) at the age of 5, 6, 7, 15 or 22 weeks in WT mice and PLN-R14 ^Δ/Δ^ mice subjected to vehicle or early or late PLN-ASO administration (*n* = 6 per group, except *n* = 4 for 5-, 6- and 7-week-old WT mice and 7-, 15- and 22-week-old ASO-late mice). Data including individual values are shown in Figure S2. Data are shown as mean ± SD. * *p* < 0.05 vs. age-matched WT mice, ^#^ *p* < 0.05 vs. age-matched PLN-R14 ^Δ/Δ^ + vehicle, ^‡^ *p* < 0.05 vs. age-matched PLN-R14 ^Δ/Δ^ + ASO-early (two-way ANOVA followed by Tukey’s post hoc test). Data of 5- to 7-week-old WT mice are derived from Eijgenraam et al. [15]. (**E**) Images of short-axis M-mode recordings of 7-week-old WT mice and PLN-R14 ^Δ/Δ^ mice receiving vehicle or early or late PLN-ASO treatment, representative for the data shown in (**A**–**C**) (scale bar: 1 mm). (**F**) Representative long-axis B-mode images of 7-week-old WT mice and PLN-R14 ^Δ/Δ^ mice receiving vehicle or early or late PLN-ASO treatment including left-ventricular epicardial and endocardial tracings with vectors indicating the direction and magnitude of wall motion, which is quantified in (**D**) (scale bar: 1 mm).

Serial echocardiographic measurements were performed to assess cardiac morphology and function. At 5 weeks of age (upon initiation of early PLN-ASO treatment), end-diastolic dimensions were not significantly different between groups, but PLN-R14 ^Δ/Δ^ mice had impaired LV contractility as evidenced by significantly increased end-systolic diameter with decreased FS and global longitudinal strain (GLS) (Figure 2A–D). Furthermore, at the start of late PLN-ASO administration at 6 weeks of age, LV end-diastolic and end-systolic diameters were significantly increased, and FS and GLS were significantly reduced in PLN-R14 ^Δ/Δ^ mice as compared to age-matched WT mice. In order to directly compare vehicle and early and late PLN-ASO groups, echocardiography was performed in all mice at 7 weeks of age (Figure 2E,F). As mentioned above, two PLN-R14 ^Δ/Δ^ mice of the ASO-late group suffered sudden cardiac death at anesthetization and could not be analyzed. Expectedly, at this age, vehicle-treated PLN-R14 ^Δ/Δ^ mice showed greatly increased ventricular dimensions and severely impaired contractile function compared to WT controls (Figure 2). Both early and late PLN-ASO treatment resulted in less pronounced LV dilatation, and preserved FS and GLS. After PLN-R14 ^Δ/Δ^ mice receiving vehicle injections reached a humane endpoint, two additional echocardiograms of WT and PLN-ASO-treated PLN-R14 ^Δ/Δ^ mice were made at the age of 15 and 22 weeks, followed by termination for tissue analyses. Reduced contractility was observed in the early treatment group at 15 and 22 weeks of age, while ASO-late mice had significantly worse FS and GLS and greater LV dimensions than WT mice and early treated PLN-R14 ^Δ/Δ^ mice (Figure 2C,D). Despite the functional deficits, these parameters remained stable until sacrifice. Echocardiographic data per time point are shown in Figure S2.

### 2.3. Cardiac Remodelling Is Attenuated by Knockdown of PLN

After in vivo analyses, mice were terminated, and heart tissues were isolated to determine the effect of PLN-ASO treatment on cardiac remodeling. Additionally, we collected heart tissues from 5-, 6- and 8-week-old WT mice and 5- and 6-week-old PLN-R14 ^Δ/Δ^ mice to compare the hearts of PLN-ASO-treated mice to WT mice of the same age and PLN-R14 ^Δ/Δ^ mice of the age of treatment initiation. The two mice of the ASO-late group that died prematurely during anesthesia were excluded from subsequent analyses.

**Figure 3:**
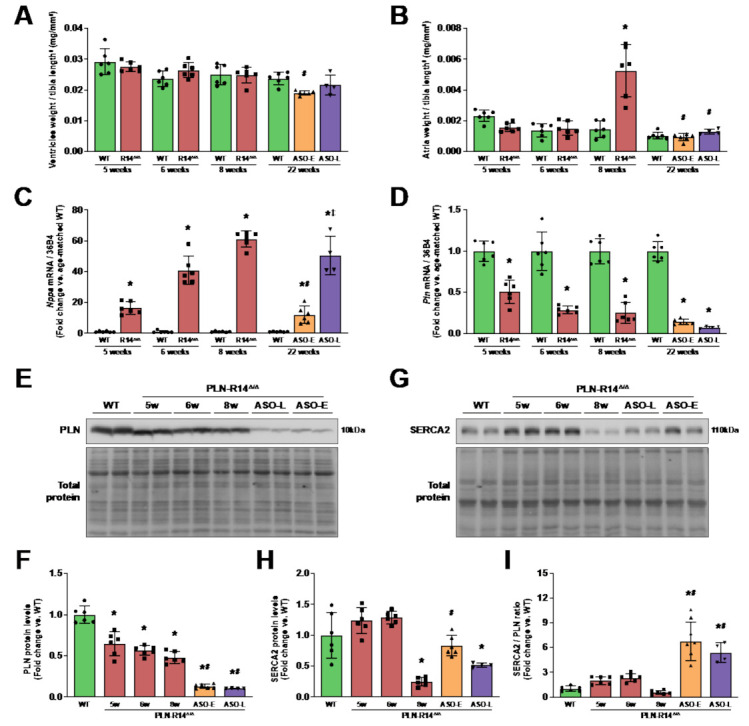
Congestive heart failure was inhibited by knockdown of PLN. (**A**,**B**) Biventricular (**A**) and atrial (**B**) weights of WT mice and vehicle-treated or early or late ASO-treated PLN-R14 ^Δ/Δ^ mice sacrificed at the age of 5, 6, 8 or 22 weeks, normalized for tibia length to the power 3. (**C**,**D**) Left-ventricular mRNA levels of *Nppa* (natriuretic peptide A; ANP) (**C**) and *Pln* (phospholamban; PLN) (**D**) normalized to housekeeping gene *Rplp0* (ribosomal protein, large, P0; 36B4) mRNA levels in WT mice and vehicle-treated or early or late ASO-treated PLN-R14 ^Δ/Δ^ mice at the age of 5, 6, 8 or 22 weeks, shown as (log2) fold change compared to age-matched WT mice. (**E**) Left-ventricular PLN protein (upper panel) and total protein levels (lower panel) in WT mice and vehicle-treated or early or late ASO-treated PLN-R14 ^Δ/Δ^ mice at the age of 5, 6, 8 or 22 weeks. (**F**) PLN protein levels are normalized to total protein levels and quantified as fold change compared to WT mice. (**G**) Left-ventricular SERCA2 protein (upper panel) and total protein levels (lower panel) in WT mice and vehicle-treated or early or late ASO-treated PLN-R14 ^Δ/Δ^ mice at the age of 5, 6, 8 or 22 weeks. (**H**) SERCA2 protein levels are normalized to total protein levels and quantified as fold change compared to WT mice. (**I**) Ratio of SERCA2 over PLN protein levels, presented as fold change compared to WT mice. *n* = 6 per group, except *n* = 4 for PLN-R14 ^Δ/Δ^ + ASO-late. Full blot images are shown in Figure S4 and S5. Data are shown as mean ± SD. *p < 0.05 vs. (age-matched) WT mice, ^#^ *p* < 0.05 vs. 8-week-old PLN-R14 ^Δ/Δ^ + vehicle, ^‡^ *p* < 0.05 vs. PLN-R14 ^Δ/Δ^ + ASO-early (two-way ANOVA followed by Tukey’s post hoc test).

Expectedly, while ventricle weights were similar between groups, PLN-R14 ^Δ/Δ^ mice had significantly increased atrial weights at 8 weeks of age as compared to age-matched WT mice (Figure 3A,B). Atrial weights were not yet increased in PLN-R14 ^Δ/Δ^ mice at the age of 5 or 6 weeks, showing that atrial hypertrophy was not present at treatment initiation. Atrial weights were similar to age-matched controls in both early and late treatment groups at the end of the experiment (22 weeks), indicating that PLN-ASO administration prevented cardiac volume overload (Figure 3A,B) [20].

In line with functional findings, LV gene expression levels of *Nppa*, encoding natriuretic peptide A (ANP), a well-established marker for heart disease [21], progressively increased with HF progression in PLN-R14 ^Δ/Δ^ mice to 16-, 40- and 60-fold elevations in 5-, 6- and 8-week-old mice, respectively (Figure 3C). Early and late PLN-ASO treatment partially relieved cardiac stress as 22-week-old PLN-R14 ^Δ/Δ^ hearts showed only a 12- and 50-fold increase in *Nppa* mRNA levels, respectively, as compared to WT mice of the same age.

To confirm the predicted PLN knockdown after PLN-ASO administration, *Pln* gene expression and PLN and SERCA2 protein levels were determined. Since WT mice had similar LV PLN protein levels between 5 and 22 weeks of age (Figure S3), WT mice were randomly selected from every age and combined as a single control group for Western blot analyses. Relative to WT mice, PLN-R14 ^Δ/Δ^ mice demonstrated progressively decreasing LV *Pln* mRNA and PLN protein levels over time (Figure 3D–F), which may be partially explained by loss of cardiomyocytes, but PLN is also frequently downregulated in HF [22,23]. SERCA2 protein expression was strongly reduced only at 8 weeks of age, which resulted in a low SERCA2/PLN ratio, likely representing end-stage HF (Figure 3G–I). PLN-ASO administration greatly reduced *Pln* gene expression (~2-fold) and PLN protein levels (~4-fold) in both early and late treatment groups as compared to 8-week-old vehicle-treated PLN-R14 ^Δ/Δ^ mice, whereas SERCA2 levels almost normalized, leading to a very high ratio of SERCA2 over PLN.

**Figure 4:**
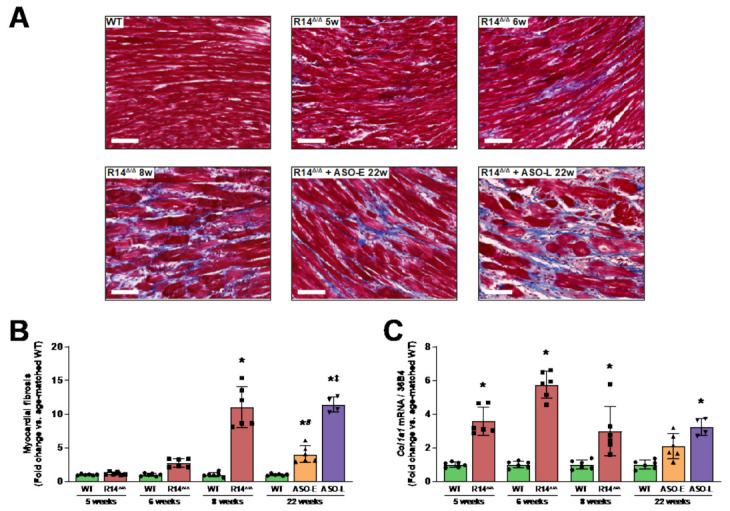
Myocardial fibrosis formation was attenuated upon PLN-ASO administration. (**A**,**B**) Representative images of Masson’s trichrome-stained cardiac tissue sections (scale bar: 70 μm) (**A**) and quantification of myocardial fibrosis in WT mice and vehicle-treated or early or late ASO-treated PLN-R14 ^Δ/Δ^ mice at the age of 5, 6, 8 or 22 weeks, depicted as fold change compared to age-matched WT mice (**B**). (**C**) Left-ventricular mRNA levels of *Col1a1* (collagen, type I, alpha I; COL1A1) normalized to housekeeping gene *Rplp0* (36B4) mRNA levels in WT mice and vehicle-treated or early or late ASO-treated PLN-R14 ^Δ/Δ^ mice at the age of 5, 6, 8 or 22 weeks, shown as fold change compared to age-matched WT mice. *n* = 6 per group, except *n* = 4 for PLN-R14 ^Δ/Δ^ + ASO-late. Data are shown as mean ± SD. * *p* < 0.05 vs. age-matched WT mice, ^#^ *p* < 0.05 vs. 8-week-old PLN-R14 ^Δ/Δ^ + vehicle, ^‡^ *p* < 0.05 vs. PLN-R14 ^Δ/Δ^ + ASO-early (two-way ANOVA and Tukey’s post hoc test).

Subsequently, the amount of myocardial fibrosis was quantified in Masson’s trichrome-stained ventricular sections (Figure 4A). Expectedly, PLN-R14 ^Δ/Δ^ hearts showed an increased amount of collagen deposition over time, which augmented up to an 11-fold increase in comparison to WT mice by 8 weeks of age (Figure 4B). Early PLN-ASO administration strongly reduced formation of myocardial fibrosis in PLN-R14 ^Δ/Δ^ mice (~4-fold at 22 weeks), whereas late PLN-ASO administration resulted in collagen deposition at 22 weeks comparable to 8-week-old vehicle-treated PLN-R14 ^Δ/Δ^ mice. Whether this level of fibrosis was already reached at early stages of ASO-late treatment or attenuated but progressive fibrotic deposition has occurred, is not clear. LV gene expression levels of collagen type I alpha 1 (*Col1a1*) corroborated the histological findings, and the elevated collagen expression levels do indicate that strongly attenuated fibrotic processes are still ongoing at 22 weeks of age in the ASO-late group (Figure 4C).

### 2.4. PLN Protein Aggregates Are Eliminated by PLN-ASO Therapy

**Figure 5:**
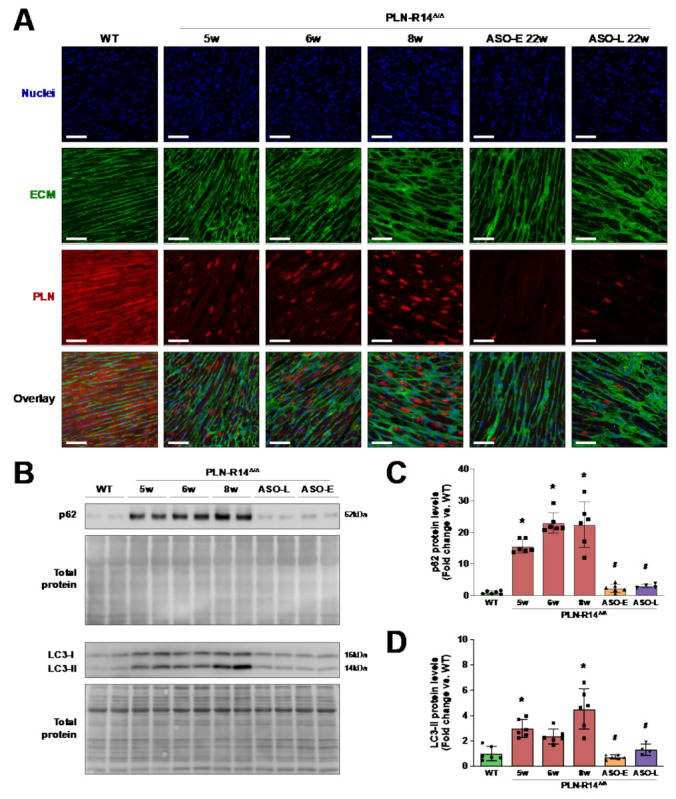
PLN protein aggregates were eliminated by PLN-ASO therapy. (**A**) Representative fluorescence images of WT mice and vehicle-treated or early or late ASO-treated PLN-R14 ^Δ/Δ^ mice at the age of 5, 6, 8 or 22 weeks stained for nuclei (DAPI) in blue (top), extracellular matrix (WGA) in green (second), PLN in red (third) and an overlay of all channels (bottom) (scale bar: 70 μm). (**B**) Left-ventricular p62/sequestome 1 (SQSTM1), microtubule-associated protein 1A/1B-light chain 3 (LC3) and corresponding total protein levels in WT mice and vehicle-treated or early or late ASO-treated PLN-R14 ^Δ/Δ^ mice sacrificed at the age of 5, 6, 8 or 22 weeks. (**C**,**D**) p62 (**C**) and LC3-II (**D**) protein levels are normalized to total protein levels and quantified as fold change compared to WT mice (*n* = 6 per group, except *n* = 4 for PLN-R14 ^Δ/Δ^ + ASO-late). Full blot images are shown in Figure S6 and S7. Data are shown as mean ± SD. * *p* < 0.05 vs. WT mice, ^#^ *p* < 0.05 vs. 8-week-old PLN-R14 ^Δ/Δ^ + vehicle (two-way ANOVA followed by Tukey’s post hoc test).

We have previously reported that, like in human patients [10,24], intracardiomyocyte aggregation of PLN proteins is an early hallmark of PLN-R14del cardiomyopathy in mice [15]. To determine PLN protein distribution, we performed immunofluorescent staining for PLN on cardiac sections. Indeed, PLN-R14 ^Δ/Δ^ mice demonstrated progressively abundant PLN protein aggregates between 5 and 8 weeks of age (Figure 5A). The PLN-ASO-mediated knockdown of PLN was apparent in both early and late groups by the reduced overall intensity of the PLN staining. Moreover, PLN protein aggregates were almost completely absent (>80% less than untreated PLN-R14 ^Δ/Δ^ mice), indicating that pre-existing PLN aggregates were resolved after inhibition of PLN expression. To support this finding, we determined protein levels of p62/sequestome 1 (SQSTM1), a protein that binds to ubiquitinated substrates and aids in proteasomal or autophagosomal degradation [25], and the autophagosome marker microtubule-associated protein 1 light chain 3 (LC3) [26]. LC3 becomes conjugated to lipid phosphatidylethanolamine (PE) on the surface of autophagosomes and can be detected as LC3-II, which has a faster motility on sodium dodecyl sulphate-polyacrylamide gel electrophoresis (SDS-PAGE) than the cytosolic LC3-I [27]. Significantly increased levels of p62 and the active LC3-II form were observed in 5- to 8-week-old PLN-R14 ^Δ/Δ^ hearts compared to WT mice, whereas these were almost normalized to WT levels in 22-week-old PLN-ASO treated mice (Figure 5B–D), indicating that protein homeostasis was re-established by this treatment.

## 3. Discussion

**Figure 6:**
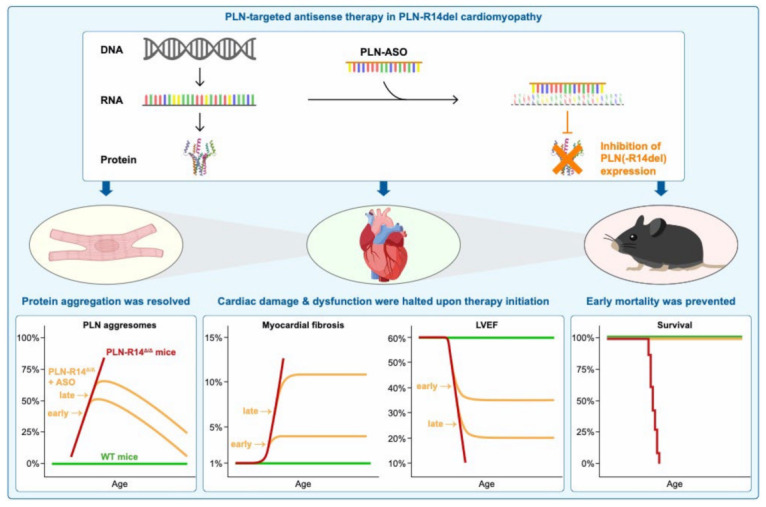
Visual summary of the main findings of this study. PLN-R14 ^Δ/Δ^ mice exhibited cardiomyopathy in an accelerated manner with a similar phenotype as human patients, including PLN protein aggregation, severe myocardial fibrosis, and HF. Upon PLN-ASO therapy, *Pln* mRNA was degraded, resulting in inhibition of PLN protein synthesis. Even when PLN-ASO was administered at an advanced stage of cardio-myopathy, PLN aggregates were cleared, and further progression of cardiac remodeling and dysfunction was halted quickly after treatment initiation. Ultimately, survival of PLN-R14 ^Δ/Δ^ mice was prolonged by at least three-fold, indicating that PLN-ASO therapy has great beneficial effects in PLN-R14del cardiomyopathy at severe disease stages.

In this study, we investigated the potential of PLN-ASO treatment to halt disease progression in PLN-R14 ^Δ/Δ^ mice with established cardiac remodeling and dysfunction. We demonstrated that PLN-ASO administration lowers PLN levels, and halts disease progression in PLN-R14 ^Δ/Δ^ mice with progressive cardiomyopathy (Figure 6). PLN-ASO therapy almost completely resolved PLN aggregates, and re-established protein homeostasis, extending maximum life span from 8 to at least 22 weeks. These results indicate that PLN-ASO therapy may have relevant beneficial effects when initiated after disease onset in PLN-R14del cardiomyopathy.

We have recently characterized the PLN-R14del mouse model, and have described that PLN-R14 ^Δ/Δ^ mice rapidly and progressively develop cardiomyopathy with severe HF between 3 and 8 weeks of age [15,16]. In addition, pre-emptive depletion of PLN using a PLN-ASO demonstrated beneficial effects in three murine models of heart disease [18]. Since patients often already have HF at primary presentation, and asymptomatic carriers are only treated after disease onset, we herein aimed to determine whether PLN-ASO administration has beneficial effects in a clinically relevant stage of advanced PLN-R14del cardiomyopathy. Our previous findings prompted us to start PLN-ASO administration in PLN-R14 ^Δ/Δ^ mice after development of moderate and severe HF, at the age of 5 and 6 weeks, respectively. Indeed, echocardiographic analysis at treatment initiation confirmed the anticipated contractile impairment and ventricular dilatation, validating that therapy was commenced in mice with existing cardiomyopathy. To compare between vehicle and early and late PLN-ASO administration at the same age, cardiac function was assessed in all mice at 7 weeks of age. Already at this age, significantly better heart function was observed in both ASO-early and ASO-late groups. While untreated PLN-R14 ^Δ/Δ^ mice die within 2 months of age due to severe HF, no further HF progression and pathological remodeling was observed in ASO-early and ASO-late groups shortly after treatment initiation, and survival was improved. In fact, a trend of improvement of systolic function was observed in the treatment groups, but a longer follow-up period would have been required to determine the significance of a potential functional improvement. Of note, the PLN-R14 ^Δ/Δ^ mouse model accurately mimics the human disease, but in an accelerated manner. In patients, the same disease characteristics develop at a much slower pace (years instead of weeks), providing a much larger therapeutic window for PLN-ASO therapy in a clinical setting.

Important findings of this study were the effects that PLN-ASO therapy had on the cellular, tissue and functional level. On the tissue level, further progression of cardiomyopathy was ceased shortly after the start of ASO administration, thereby preventing late-onset manifestations including atrial enlargement, and resulting in prolonged survival, but existing tissue damage and functional impairment could not be reversed. The patchy distribution of scar tissue indicates cardiomyocyte death and subsequent formation of replacement fibrosis, which likely cannot be reversed due to the negligible regenerative capacity of the heart [28]. Correspondingly, the earlier start of treatment in the ASO-early group resulted in better outcomes at the study endpoint as compared to late treatment, underlining timely therapy initiation. Early- and late-treated PLN-R14 ^Δ/Δ^ mice demonstrated a similar ASO-mediated reduction in PLN levels, but at the start of late PLN-ASO therapy (7 weeks of age), PLN-R14 ^Δ/Δ^ mice had developed worse HF than when treatment was started in the ASO-early group (6 weeks of age), resulting in worse LV function, higher ANP levels and more fibrosis formation in the ASO-late group at the study endpoint. On the other hand, the most striking result was observed within the cardiomyocytes. For the majority of proteins, there is a continuous balance of protein synthesis and degradation to replace old, damaged or non-functional proteins with new functional proteins [29]. This process of protein turnover is very dynamic, and ASOs can act on this process by adapting mRNA splicing or targeting mRNAs for degradation or translational arrest, thereby decreasing protein production and ultimately resulting in decreased protein levels [17]. Interestingly, PLN-ASO administration not only reduced PLN expression, but also resolved PLN protein aggregates, and normalized autophagy markers. This is in line with a previous study that demonstrated a reduction in existing TTR deposits in a transgenic mouse model of TTR-mediated amyloidosis (ATTR amyloidosis) after siRNA-mediated knockdown of TTR [30]. Together, this suggests that protein aggregation is the result of an imbalance between protein synthesis and degradation, and this balance may be restored by inhibition of synthesis of pathogenic or aggregation-prone proteins. In case of a primary genetic defect, such as PLN-R14del, understanding and targeting the molecular mechanisms that lead to cardiomyopathy is key for successful therapy [31]. Although it is yet unclear if protein aggregates are a cause or consequence of this disease, PLN aggregation clearly is a pathological biomarker as it preceded other cardiac abnormalities [15], and was resolved along with attenuation of HF. Along the same line of reasoning, Feyen et al. recently demonstrated that knockdown of endoplasmic reticulum (ER) stress sensors exaggerated contractile dysfunction of PLN-R14del-induced pluripotent stem cell-derived cardiomyocytes, while stimulation of the unfolded protein response improved contractility and force development to amplitudes that were similar to cardiomyocytes derived from healthy donors [32].

We acknowledge several limitations in the current study. Validation of PLN inhibition after PLN-ASO administration was limited by the different ages and stages of cardiomyopathy of the experimental groups. Expression of calcium regulatory proteins is commonly altered in heart disease [22], and, similarly, PLN expression gradually decreased over time in PLN-R14 ^Δ/Δ^ mice. Ideally, PLN knockdown should be determined at the same age in vehicle- and PLN-ASO-treated mice with comparable cardiac function to exclude the influence of HF on PLN expression, but this is not possible with the current experimental design, in which we examined the survival of the mice. Nevertheless, at the end of this study, PLN protein levels (by itself and relatively to SERCA2) were greatly reduced by PLN-ASO treatment as compared to vehicle, while heart function was preserved. An intrinsic limitation to the use of ASOs is the need for repeated administration to maintain mRNA depletion, and possibility of accumulation and toxicity in other organs such as the liver and kidneys that clear these compounds from the system [17]. Efforts have been made to modify ASO structure to increase potency in target organs and reduce systemic toxicity [17]. In addition, as PLN plays an important role in cardiac physiology, caution has to be taken when reducing PLN levels. In contrast to PLN knock-out mice that exert improved LV contractility [33], the T116G or Leu39Stop variant of the PLN gene was found to result in a truncated protein that was associated with DCM in homozygous carriers [34]. Taken together, further research is warranted to determine the optimal dosing regimen for sufficient efficacy without major side effects, and, ideally, an R14del-specific ASO should be considered.

In conclusion, the results of this study implicate that PLN-ASO exerts beneficial effects in PLN-R14del cardiomyopathy when administered after disease onset. Although existing dysfunction and organ damage including cardiac fibrosis could not be reversed, PLN expression and aggregation were diminished in the remaining cardiac tissue, which resulted in prevention of further disease progression and an extension of maximum life span of PLN-R14 ^Δ/Δ^ mice. Since the PLN-R14del pathogenic variant is the root cause of this heart disease, elimination of PLN expression can be considered a very promising therapeutic approach, providing a great example of a potential personalized medicine for PLN-R14del carriers.

## 4. Materials and Methods

An expanded methods section is provided in the Supplementary Materials.

### 4.1. Experimental Animals

All animal experiments were approved by the animal ethical committee of the University of Groningen (permit numbers AVD10500201583, IVD1583-02-001 and IVD1583-02-006), performed conform the guidelines from Directive 2010/63/EU of the European Parliament on the protection of animals used for scientific purposes, and reported following the ARRIVE guidelines for reporting animal research [35]. Cardiac analyses and euthanasia were performed under continuous anesthesia of 2% isoflurane (TEVA Pharmachemie, Haarlem, The Netherlands) mixed with oxygen, administered via an aerial dispenser. Heart and respiration rates were continuously monitored throughout all procedures to ensure adequate levels of anesthesia. Generation and characterization of PLN-R14del mice has been previously published [16].

### 4.2. Study Design

To study the effect of PLN inhibition in established HF due to the PLN-R14del pathogenic variant, male and female PLN-R14 ^Δ/Δ^ mice (*n* = 6 per group in total) were randomly subjected based on sex (50/50% male/female) to subcutaneous (sc) injections of 50 mg/kg ASO (5′-GCATATCAATTTCCTG-3′, Ionis Pharmaceuticals, Carlsbad, CA, USA) that targets murine *Pln* pre-mRNA for degradation, starting at the age of 5 (moderate HF, “early” treatment) or 6 weeks (severe HF, “late” treatment) (Figure 1A). Generation of the PLN-ASO, and validation of its efficacy and safety have been reported elsewhere [18]. Since our previous study found no differences between administration with scrambled ASO or vehicle [18], the control group received vehicle (0.9% NaCl solution) injections. The dosing scheme included an initial loading phase of 3 injections of 50 mg/kg PLN-ASO in the first week of treatment followed by a maintenance phase of weekly injections of 50 mg/kg. The dosing regimen was predicted to result in a ~80% reduction in *Pln* mRNA in cardiac tissue at steady-state [18]. Echocardiography was performed upon treatment initiation (5 or 6 weeks of age), and at the age of 7 and 15 weeks. Mice were monitored for their maximum life span or until a maximum of 22 weeks of age, after which final echocardiographic analysis was performed, mice were terminated, and tissues were collected for histological and molecular analyses. In addition, 6 WT mice were subjected to echocardiography at the age of 15 and 22 weeks, after which tissues were collected. To reduce the anesthesia burden, echocardiographic data of 5- to 7-week-old WT mice from our previous study were included as a reference of normal values for those ages [15]. Finally, hearts were isolated from another 6 WT and PLN-R14 ^Δ/Δ^ mice at 5, 6 or 8 weeks of age. Data acquisition and analyses were performed in a blinded fashion.

### 4.3. Echocardiography

Transthoracic echocardiography was performed using a Vevo imaging station and a Vevo 3100 preclinical imaging system, equipped with a 40-MHz MX550D linear array transducer (FUJIFILM VisualSonics, Toronto, Canada). General techniques have been reported before [36]. Vevo LAB software was used to assess LV morphology and function, and to evaluate GLS using speckle-tracking. Data acquisition and analysis were executed in line with the recommendations of the European Society of Cardiology (ESC) Working Group on Myocardial Function [37].

### 4.4. Euthanasia

Euthanasia was performed as previously reported [38]. Briefly, after mice were anesthetized, the abdomen was opened, the aorta was cut, and the circulation was perfused with saline. The heart was quickly excised, rinsed in 1 M KCl solution to arrest the heart in diastole, weighed and dissected. A transverse mid-slice was fixed overnight in 4% buffered formaldehyde solution (10% formalin, Klinipath, Duiven, The Netherlands) for histology. Remaining LV tissues were snap-frozen in liquid nitrogen for molecular analyses. Tibias were collected from the right hind leg for indexing heart weights by tibia length to the power 3 to normalize for body size [39].

### 4.5. Quantitative Polymerase Chain Reaction

RNA was isolated from snap-frozen LVs using TRI Reagent (Sigma-Aldrich, Saint Louis, MO, USA). cDNA synthesis was performed using the QuantiTect RT kit (Qiagen, Hilden, Germany). Gene expression was determined by qPCR using iQ SYBR green supermix (Bio-Rad, Hercules, CA, USA) as previously described [40]. mRNA levels were normalized to reference gene values of a component of the large 60 S ribosomal subunit (*Rplp0*, encoding 36B4) using CFX Manager software (version 3.0, Bio-Rad). Calculated values are shown relative to age-matched WT mice. Primer sequences are listed in Table S1.

### 4.6. Western Blot

Proteins were isolated from snap-frozen LVs using radioimmunoprecipitation assay (RIPA) lysis buffer as previously described [41]. Protein samples were centrifuged at 12,000× *g* for 10 min at 4 °C, and the supernatant containing soluble proteins was collected. Since the PLN-R14del pathogenic variant causes PLN to form RIPA-insoluble aggregates, the remaining pellets were dissolved in 8 M urea solution and combined with the RIPA lysates. Protein concentrations were determined using a Pierce bicinchoninic acid (BCA) protein assay kit (Thermo Scientific, Waltham, MA, USA). Equal amounts of proteins were denatured, separated by SDS-PAGE, and transferred onto Immun-Blot polyvinylidene fluoride (PVDF) membranes (Bio-Rad). After overnight incubation at 4 °C with a primary antibody, membranes were incubated with an appropriate horseradish peroxide (HRP)-linked secondary antibody, and detection was performed using Western Lightning Ultra chemiluminescence reagent (PerkinElmer, Waltham, MA, USA) and an ImageQuant LAS 4000 digital imaging system (GE Healthcare, Chicago, IL, USA). The density of each band was quantified using Image Studio Lite software (version 5.2.5, LI-COR Biosciences, Lincoln, NE, USA), and normalized to total protein levels as determined using Revert 700 Total Protein Stain (LI-COR Biosciences). Calculated values are shown relative to WT mice. Antibodies are listed in Table S2 and S3.

### 4.7. Histological Analyses

Formalin-fixed cardiac transverse mid-slices were dehydrated, cleared, infiltrated with and embedded in histological paraffin wax (Klinipath), and subsequently cut into 4-μm thick sections. Masson’s trichrome stain was performed to detect collagen deposition as a measurement of fibrosis as previously described [42]. Stained sections were imaged using a NanoZoomer 2.0-HT digital slide scanner (Hamamatsu Photonics, Hamamatsu, Japan), and the percentage of myocardial fibrosis was quantified using the Positive Pixel Count v9 algorithm of Aperio’s ImageScope software (Leica Microsystems, Wetzlar, Germany). The amount of myocardial fibrosis is shown as fold change to the age-matched WT group.

Immunofluorescence was performed using an anti-PLN antibody (clone 2D12, Invitrogen, Carlsbad, CA, USA), which has been shown to stain PLN-R14del proteins [10,15,16,24], labelled with Alexa Fluor 555 (red) using an APEX antibody labelling kit (Invitrogen). Sections were co-stained with fluorescein isothiocyanate (FITC)-conjugated wheat germ agglutinin (WGA, Sigma-Aldrich) to stain extracellular matrix (ECM) green, and 4′,6-diamidino-2-phenylindole (DAPI, Vector Laboratories, Burlingame, CA, USA) to stain nuclei blue [43].

### 4.8. Statistical Analyses

For statistical comparisons of survival curves, log-rank tests were performed. Other data are presented as means ± standard deviations (SD) and are analyzed by two-way analysis of variance (ANOVA) tests, followed by Tukey’s post hoc tests to correct for multiple comparisons. Only comparisons between all PLN-R14 ^Δ/Δ^ groups and (age-matched) WT mice, ASO-treated PLN-R14 ^Δ/Δ^ mice (early and late) versus 8-week-old untreated PLN-R14 ^Δ/Δ^ mice, and ASO-early versus ASO-late are depicted. *p*-values < 0.05 were considered statistically significant. SPSS Statistics software (version 27, IBM, Armonk, NY, USA) was used for all statistical analyses.

## Data Availability

All data underlying this work are available in the article and supplementary data.

## Supplementary Materials

The following supporting information can be downloaded at: www.mdpi.com/1422-0067/23/5/2427/s1.
