# Supplementary material for: Antisense Therapy Attenuates Phospholamban p.(Arg14del) Cardiomyopathy in Mice and Reverses Protein Aggregation
Source: Int J Mol Sci. 2022 Feb 22;23(5):2427. doi: 10.3390/ijms23052427 (PMC8909937; doi:10.3390/ijms23052427)
Supplement: Supplementary file 1 [file ijms-23-02427-s001.zip › ijms-1575635-supplementary.pdf]

— Supplementary Material —

Antisense Therapy Attenuates Phospholamban p.(Arg14del) Cardiomyopathy in Mice and Reverses Protein Aggregation

Tim R. Eijgenraam [1], Nienke M. Stege [1], Vivian Oliveira Nunes Teixeira [1], Remco de Brouwer [1,2], Elisabeth M. Schouten [1], Niels Grote Beverborg [1], Liu Sun [1], Daniela Später [3], Ralph Knöll [3], Kenny M. Hansson [3], Carl Amilon [4], David Janzén [5], Steve T. Yeh [6], Adam E. Mullick [6], Peter van der Meer [1], Rudolf A. de Boer [1,†] and Herman H. W. Silljé [1,†,*]

[1] University Medical Center Groningen, Department of Cardiology, University of Groningen, Antonius Deusinglaan 1, 9713 AV Groningen, The Netherlands; t.r.eijgenraam@umcg.nl (T.R.E.); n.m.stege@umcg.nl (N.M.S.); v.oliveira.nunes.teixeira@umcg.nl (V.O.N.T.); r.de.brouwer@umcg.nl (R.d.B.); e.m.schouten@umcg.nl (E.M.S.); n.grote.beverborg@umcg.nl (N.G.B.); l.sun@umcg.nl (L.S.); p.van.der.meer@umcg.nl (P.v.d.M.); r.a.de.boer@umcg.nl (R.A.d.B.)

[2] Netherlands Heart Institute, Moreelsepark 1, 3511 EP Utrecht, The Netherlands

[3] Bioscience Cardiovascular, Research and Early Development, Cardiovascular, Renal and Metabolism (CVRM), BioPharmaceuticals R&D, AstraZeneca, Pepparedsleden 1, 431 50 Gothenburg, Sweden; daniela.spaeter@astrazeneca.com (D.S.); ralph.knoell@astrazeneca.com (R.K.); kenny.m.hansson@astrazeneca.com (K.M.H.)

[4] Projects, Research and Early Development, Cardiovascular, Renal and Metabolism, BioPharmaceuticals R&D, AstraZeneca, Pepparedsleden 1, 431 50 Gothenburg, Sweden; carl.amilon@telia.com

[5] Drug Metabolism and Pharmacokinetics, Research and Early Development, Cardiovascular, Renal and Me-tabolism, BioPharmaceuticals R&D, AstraZeneca, Pepparedsleden 1, 431 50 Gothenburg, Sweden; david.janzen@astrazeneca.com

[6] Ionis Pharmaceuticals, 2855 Gazelle Court, Carlsbad, CA 92010, USA; syeh@ionisph.com (S.T.Y.); amullick@ionisph.com (A.E.M.)

* Correspondence: h.h.w.sillje@umcg.nl; Tel.: +31-(0)-50-361-5338

† The last two authors contributed equally to this work.

1. Supplementary methods

1.1. Experimental animals

Generation and characterization of mice carrying the phospholamban (PLN) p.(Arg14del) pathogenic variant (R14del) have been described before [1]. In summary, a C57Bl6/N mouse line was generated, in which the third exon of the murine *Pln* gene, which contains the coding region for the PLN protein, was flanked by *loxP* sites (*floxed*) and followed by a third exon of the murine *Pln* gene with the c.40_42delAGA pathogenic variant (performed by PolyGene, Switzerland). To delete the *floxed* region, these mice were bred with mice expressing the *Cre* enzyme in the germline under the control of the hypoxanthine-guanine phosphoribosyltransferase (*Hprt*) promoter enhancer, replacing the murine wild-type (WT) *Pln* exon-3 with the murine R14del *Pln* exon-3 in the resulting offspring (PLN-R14$^{\Delta/+}$). The offspring was backcrossed into a C57Bl6/J background. PLN-R14$^{\Delta/+}$ mice were crossbred to generate PLN-R14$^{\Delta/\Delta}$ mice. Mice were housed per nest and per sex on a 12 h light / 12 h dark cycle with *ad libitum* access to chow and water. Cardiac analyses and euthanasia were performed under continuous anesthesia of 2% isoflurane (TEVA Pharmachemie, the Netherlands) mixed with oxygen, administered via an aerial dispenser. Heart and respiration rates were continuously monitored throughout all procedures. When monitoring maximum survival, mice were terminated at a predefined humane endpoint (indicating severe heart failure) to prevent excessive discomfort. For this study, the humane endpoint was defined by presence of lethargy and dyspnea, with a maximum of 20% weight loss. Since we had no prior evidence for sex differences, both male and female mice (50-50%) have been used.

1.2. Genotyping

For genotyping of PLN-R14del mice, DNA was isolated from ear cuts using the prepGEM Universal kit (ZyGEM Corporation, New-Zealand) following manufacturer's instructions for DNA isolation from solid tissue. Ear cuts were incubated with ORANGE+ buffer, Histosolv and prepGEM in demiwater at 52°C for 5 min, 75°C for 10 min and 95°C for 3 min using a T100 thermal cycler (Bio-Rad, CA, USA) to extract DNA. To identify genotypes, quantitative polymerase chain reaction (qPCR) analysis was performed using iQ SYBR green supermix (Bio-Rad) according to the manufacturer's instructions. DNA isolated from ear cuts was mixed 1:50 with 0.5 µM of forward (5'-ACCCAGGACAGTGAGAC-3') and reverse (5'-GCTTTGCAGCAGCTCGTTC-3') primers (Bio-Rad) and iQ SYBR green supermix (Bio-Rad). The qPCR reaction was performed at 95°C for 5 min, 35 cycles of 95°C for 30 sec, 55°C for 15 sec and 69°C for 30 sec, followed by a melt curve from 81°C to 86°C with increments of 0.2°C every 5 sec using a CFX384 Touch real-time PCR detection system (Bio-Rad). Since after *Cre-loxP*-mediated recombination one *loxP* site (consisting of 117 base pairs, including the 34 base pairs of the *loxP* site) remains present in the R14del *Pln* allele, presence of the WT and/or mutant allele is identified based on the size of the qPCR product (107 base pairs for the WT allele, 224 base pairs for the mutant allele), which can be distinguished based on the temperature of the melt peak (~83°C *vs.* ~85°C) using CFX Manager software (version 3.0; Bio-Rad). Validity of the melt peak identification has been confirmed by separation of qPCR products using agarose gel electrophoresis (2% agarose (Invitrogen, CA, USA) dissolved in Tris-acetate-ethylenediaminetetraacetic acid (EDTA) (TAE) buffer (40 mM Tris (Sigma-Aldrich, MO, USA), 20 mM glacial acetic acid (Merck Millipore, MA, USA) and 1 mM EDTA (Merck Millipore) in distilled water) with 0.5 µg/ml ethidium bromide (Sigma-Aldrich).

1.3. Echocardiography

Transthoracic echocardiography was performed using a Vevo imaging station (FUJIFILM VisualSonics, Canada) and a Vevo 3100 preclinical imaging system (FUJIFILM VisualSonics), equipped with a 40-MHz MX550D linear array transducer (FUJIFILM VisualSonics). Prior to echocardiographic imaging, mice were anesthetized (2% isoflurane (TEVA Pharmachemie) mixed with oxygen, administered via an aerial dispenser) and the hair was removed from the thorax using a commercially available topical depilation agent with potassium thioglycolate (Veet). Mice were fixed in supine position on the temperature-maintained (37°C) platform of the Vevo imaging station (FUJIFILM

VisualSonics) over the integrated electrode pads to monitor the heart and respiration rates. Left-ventricular (LV) parasternal long axis B-mode recordings and short axis M-mode recordings were obtained at the mid-papillary level. Vevo LAB software (version 5.5.0; FUJIFILM VisualSonics) was used for image analysis. Parasternal long axis B-mode recordings were used for speckle-tracking analysis to evaluate global longitudinal strain (GLS) by outlining the epicardial and endocardial borders. Short axis M-mode recordings were used to determine heart rate, LV end-diastolic internal diameter (LVIDd), LV end-systolic internal diameter (LVIDs) and fractional shortening (FS) by outlining the epicardial and endocardial borders using the LV Trace tool. For all measurements, three subsequent cardiac cycles, unaffected by respiration, were analyzed.

1.4. Quantitative polymerase chain reaction

RNA was isolated from snap-frozen LV tissues using TRI Reagent (Sigma-Aldrich) according to the manufacturer's protocol. Snap-frozen LV tissues were mechanically disrupted, and approximately 25 mg of powdered LV tissue was homogenized in 1 ml of TRI Reagent (Sigma-Aldrich) using a TissueLyser LT (Qiagen, Germany) at 50 Hz for 5 min. After incubation for 5 min at room temperature to ensure complete dissociation of nucleoprotein complexes, phases were separated by thoroughly mixing with 0.2 ml chloroform (Merck Millipore), incubation for 2 min at room temperature, and centrifugation at 12,000 *g* for 15 min at 4°C. The RNA-containing colorless upper aqueous phase was isolated, and RNA was precipitated by mixing with 0.5 ml 2-propanol (Biosolve, the Netherlands) and incubation for 10 min at room temperature, followed by centrifugation at 12,000 *g* for 10 min at 4°C. The supernatant was discarded, and the RNA pellet was washed twice by mixing with 1 ml 75% ethanol (Merck Millipore) and centrifugation at 12,000 *g* for 5 min at 4°C. The supernatant was removed, and the RNA pellet was air-dried before dissolving in RNase-free water. RNA concentrations were determined by spectrophotometry using a NanoDrop 2000 spectrophotometer (Thermo Scientific, MA, USA).

Next, cDNA synthesis was performed using QuantiTect reverse transcription (RT) kit (Qiagen) following manufacturer's instructions. For every sample, 500 ng of isolated total RNA was incubated with gDNA wipe-out buffer (Qiagen) at 42°C for 2 min to remove any contaminating genomic DNA. After gDNA elimination, the purified RNA samples were converted to cDNA by reverse transcription by adding Quantiscript reverse transcriptase (Qiagen) and RT primer mix (Qiagen) in Quantiscript RT buffer (Qiagen). The RT reaction was performed at 42°C for 15 min and was subsequently inactivated at 95°C for 3 min using a T100 thermal cycler (Bio-Rad).

Gene expression levels were determined by qPCR analysis using iQ SYBR green supermix (Bio-Rad) according to the manufacturer's instructions. Duplicates of 7.5 ng cDNA were mixed with 750 nM forward and reverse primers and iQ SYBR green supermix (Bio-Rad). The qPCR reaction was performed at 95°C for 3 min followed by 35 cycles of 95°C for 15 sec and 60°C for 30 sec using a CFX384 Touch real-time PCR detection system (Bio-Rad). Gene expression was quantified by correcting for reference gene values of one of the components of the large 60S ribosomal subunit (*Rplp0*, encoding 36B4) using CFX Manager software (version 3.0; Bio-Rad), and the calculated values were expressed relative to the age-matched WT group. Primer sequences can be found in Table S1.

1.5. Western blot

For protein isolation, snap-frozen LV tissues were mechanically disrupted, and approximately 25 mg of powdered tissue was homogenized in 0.2 ml ice-cold radioimmunoprecipitation assay (RIPA) lysis buffer (50 mM Tris (Sigma-Aldrich), 1.0% IGEPAL CA-630 (Sigma-Aldrich), 0.5% sodium deoxycholate (Sigma-Aldrich), 0.1% sodium dodecyl sulfate (SDS) (Sigma-Aldrich) and 150 mM NaCl (Merck Millipore) in distilled water) freshly supplemented with 4% cOmplete protease inhibitor (PI) cocktail (Roche Diagnostics, Switzerland), 1% phosphatase inhibitor cocktail 3 (Sigma-Aldrich), 15 mM sodium orthovanadate (Sigma-Aldrich), and 1 mM phenylmethylsulphonyl fluoride (PMSF) (Roche Diagnostics) using a TissueLyser LT (Qiagen) at 50 Hz for 5 min. After centrifugation at 12,000 *g* for 10 min at 4°C, the supernatant containing solubilized proteins was collected. The remaining pellet, which

contains insoluble PLN proteins, was dissolved in 50 µl urea solution (8 M urea (Sigma-Aldrich), 0.1 M NaH_2PO_4 (Merck Millipore) and 0.01 M Tris-HCl (Sigma-Aldrich) in distilled water) complemented with 1.25 U Pierce Universal nuclease for cell lysis (Thermo Scientific). Since protein aggregates are present in the cardiomyocytes of PLN-R14$^{\Delta/\Delta}$ mice, and it is yet unknown what the exact contents of these aggregates are, we combined the RIPA-soluble and RIPA-insoluble fractions into total protein lysates to prevent protein expression levels from being affected by protein (in)solubility.

Protein concentrations were determined using a Pierce bicinchoninic acid (BCA) protein assay kit (Thermo Scientific) according to the manufacturer's protocol. Duplicates of protein samples were mixed 1:200 (v:v) with working reagent (50 reagent A : 1 reagent B) in a flat-bottom 96-wells plate and incubated at 37°C for 30 min for the proteins to reduce Cu^{2+} to Cu^{1+} (biuret reaction), which is chelated with two BCA molecules. Absorbance of the purple-colored reaction product was measured at 562 nm using a Synergy H1 microplate reader (BioTek, VT, USA). Absorbance values were corrected for the average absorbance value of a blank sample (lysis buffer), after which protein sample concentrations were determined based on a standard curve of bovine serum albumin (BSA) samples with known concentrations using a quadratic curve fit using Gen5 software (BioTek).

Protein expression levels were determined by Western blotting. Equal amounts of proteins (5 µg for PLN, SERCA2 and LC3 or 15 µg for p62) with sample buffer (2% SDS (Sigma-Aldrich), 10% glycerol (Sigma-Aldrich), 60 mM Tris (Sigma-Aldrich), 0.3% dithiothreitol (DTT) (Sigma-Aldrich) and a spoon tip of bromophenol blue (Sigma-Aldrich) in demiwater) were denatured at 95°C for 5 min (which disrupts PLN pentamers) and separated by SDS-polyacrylamide gel electrophoresis (PAGE) on ice using hand cast gels (stacking gel: 0.1% SDS (Sigma-Aldrich), 125 mM Tris (Sigma-Aldrich), 4% acrylamide (Sigma-Aldrich), 0.3% ammonium persulfate (APS) (Sigma-Aldrich) and 0.04% tetramethylethylenediamine (TEMED) (Carl Roth, Germany) in demiwater; separation gel: 0.1% SDS (Sigma-Aldrich), 375 mM Tris (Sigma-Aldrich), 15% (for PLN and LC3), 10% (for p62) or 7.5% (for SERCA2) acrylamide (Sigma-Aldrich), 0.1% APS (Sigma-Aldrich) and 0.1% TEMED (Carl Roth) in demiwater) and electrophoresis buffer (1% SDS (Sigma-Aldrich), 250 mM Tris (Sigma-Aldrich), 1.92 M glycine (Sigma-Aldrich) in demiwater) in a mini-PROTEAN system (Bio-Rad). Separated proteins were transferred onto Immun-Blot polyvinylidene fluoride (PVDF) membranes (pore size 0.2 µm for PLN, p62 and LC3 or 0.45 µm for SERCA2; Bio-Rad) by semi-dry blotting (for 1h for PLN, p62 and LC3 or 2 h for SERCA2) using transfer buffer (25 mM Tris (Sigma-Aldrich), 192 mM glycine (Sigma-Aldrich) and 20% methanol (VWR Chemicals, PA, USA) in demiwater). Next, membranes were blocked in 5% BSA (SERVA Electrophoresis, Germany) or dry-milk (Campina, the Netherlands) in Tris-buffered saline (TBS) (150 mM NaCl (Merck Millipore) and 10 mM Tris (Sigma-Aldrich) in distilled water) with 0.1% TWEEN (polysorbate) 20 (Sigma-Aldrich) (TBST)) for a minimum of 1 h at room temperature while shaking and incubated overnight at 4°C with a primary antibody in block buffer while shaking. To remove unbound primary antibody, membranes were washed three times in TBST for 5 min while shaking, followed by 1-h incubation at room temperature with an appropriate horseradish peroxide (HRP)-linked secondary antibody in 5% dry-milk in TBST while shaking. After washing off unbound secondary antibody, detection was performed using Western Lightning Ultra chemiluminescence reagent (PerkinElmer, MA, USA) and an ImageQuant LAS 4000 digital imaging system (GE Healthcare, IL, USA). Quantification was performed using Image Studio Lite software (version 5.2, LI-COR Biosciences, NE, USA). The density of each band was normalized to total protein levels as determined using Revert 700 Total Protein Stain (LI-COR Biosciences). Calculated values are shown relative to WT mice. Antibodies are listed in Table S2 and S3.

1.6. Histological analysis

After sacrifice, a transverse mid-slice of the heart (~1 mm thick) was fixed overnight in 4% buffered formaldehyde solution (10% formalin; Klinipath, the Netherlands). Next, formalin-fixed tissues were processed using a Leica TP1020 automated tissue processor (Leica Microsystems, Germany). Samples were subjected to a dehydration series (70% ethanol (Klinipath) for 1 h, 80% ethanol for 1 h, 90% ethanol for 1 h, and three times 100% ethanol for 1 h) to remove most of the water from the

samples, and cleared from ethanol by washing three times with xylene (Klinipath) for 1 h, followed by two 1-h infiltrations with histological paraffin wax (Klinipath). Processed tissue samples were subsequently embedded in histological paraffin wax (Klinipath) using a Leica EG1150 H paraffin embedding module (Leica Microsystems). Embedded tissue slices were cut into 4-μm thick transversal sections using a Leica RM2255 microtome (Leica Microsystems), and mounted on StarFrost Adhesive silane-coated microscope slides (Waldemar Knittel Glasbearbeitungs, Germany). Sections were incubated overnight at 60°C for deparaffinization of the tissues. For complete deparaffinization and rehydration of the tissues, sections were incubated in xylene for 20 min, 100% ethanol for 10 min, 96% ethanol for 5 min, 70% ethanol for 1 min, and rinsed with distilled water before histological analysis.

Masson's trichrome stain was performed to detect collagen deposition as a measurement of fibrosis. Nuclei were stained black by incubation in Weigert's iron hematoxylin solution (1 g/L hematoxylin solution (Gill no. I; Sigma-Aldrich), 0.145 g/L $FeCl_3$ (Sigma-Aldrich) and 0.5% HCl (Merck Millipore) in distilled water) for 10 min. Sections were washed in running tap water for 10 min and rinsed with distilled water. Next, cytoplasm was stained red by incubation in Biebrich scarlet-acid fuchsin solution (0.9% Biebrich scarlet (VWR Chemicals), 0.1% acid fuchsin (Sigma-Aldrich) and 0.5% glacial acetic acid (Merck Millipore) in distilled water) for 13 min. Sections were washed twice with distilled water before differentiation in phosphomolybdic-phosphotungstic (PP) acid solution (3% phosphomolybdic acid hydrate (Alfa Aesar, MA, USA) and 2.5% phosphotungstic acid (Sigma-Aldrich) in distilled water) for 14 min. Without rinsing, sections were transferred to aniline blue solution (1.25% aniline blue (Acros Organics, Belgium) and 2% glacial acetic acid in distilled water) for 7 min to stain collagen blue. Sections were washed twice with distilled water before differentiation in 1% glacial acetic acid solution for 4 min. Subsequently, sections were rinsed with distilled water, and dehydrated in 96% ethanol for 15 sec, 100% ethanol for 2 min and xylene for 10 min. Sections were covered with DPX (a mixture of distyrene, a plasticizer (tricresyl phosphate) and xylene) neutral mounting medium (Sigma-Aldrich) and a cover slip. To quantify the amount of fibrosis, whole stained sections were automatically imaged using a NanoZoomer 2.0-HT digital slide scanner (Hamamatsu Photonics, Japan), and fibrotic area was determined using the Positive Pixel Count v9 algorithm of Aperio's ImageScope software (version 12.4.0; Leica Microsystems) with default settings, hue value 0.66 and hue width 0.2. Fibrosis fractions were quantified as a percentage of the total area of the entire stained section, and calculated as fold change compared to the age-matched WT group.

To visualize PLN protein aggregates, immunofluorescent staining for PLN was performed using a mouse monoclonal anti-PLN antibody (clone 2D12; #MA-922, Invitrogen), which has been shown to recognize (aggregated) PLN-R14del proteins [1–5], labelled with Alexa Fluor 555 (red) using an APEX antibody labelling kit (Invitrogen) according to the manufacturer's protocol. The anti-PLN antibody was loaded onto the prehydrated resin of the APEX antibody labelling tip together with the fluorescent label, and incubated for 2 h at room temperature, followed by elution of the labelled antibody. Tissue sections were washed three times in PBS (1.76 mM KH_2PO_4 (Merck Millipore), 10 mM Na_2HPO_4 (Sigma-Aldrich), 136.9 mM NaCl (Merck Millipore) and 2.68 mM KCl (Merck Millipore) in distilled water) in 5 min. Antigen retrieval was performed by incubating sections for 15 min in heated antigen retrieval buffer (10 mM Tris (Sigma-Aldrich) and 1 mM EDTA (Merck Millipore) in distilled water). After cooling down, excess antigen retrieval buffer was removed by washing three times in PBS in 5 min. Next, sections were incubated for 1 h in the dark with labelled anti-PLN antibody (1:200) to stain PLN red, and fluorescein isothiocyanate (FITC)-conjugated wheat germ agglutinin (WGA; Sigma-Aldrich) solution (2 mg/ml in PBS; 1:100) to stain extracellular matrix (ECM) green. After rinsing excess antibody three times with PBS for 10 min, sections were incubated in VECTASHIELD mounting medium with 4′,6-diamidino-2-phenylindole (DAPI; Vector Laboratories, CA, USA) for 30 min to stain nuclei blue. Sections were covered by a cover slip, which was sealed using blank nail polish, and stored at 4°C protected from light until imaging. Fluorescent imaging was done using a Leica AF6000 fluorescence imaging system (Leica Microsystems).

2. Supplementary figures

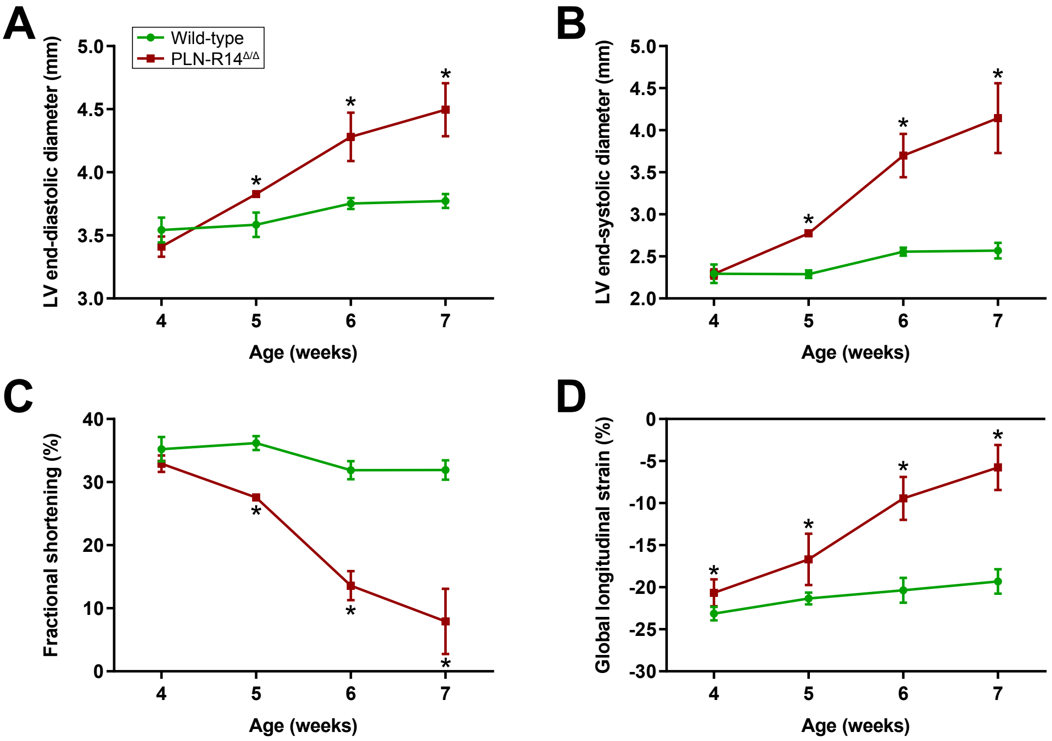

Figure S1. PLN-R14$^{\Delta/\Delta}$ mice rapidly and progressively develop dilated cardiomyopathy with heart failure between 4 and 7 weeks of age. (**A-D**) Serial LV end-diastolic (**A**) and end-systolic (**B**) internal diameters, percentage of fractional shortening (**C**) and percentage of global longitudinal strain (**D**) of WT and PLN-R14$^{\Delta/\Delta}$ mice at the age of 4, 5, 6 and 7 weeks, assessed using echocardiography (n=4 per group). Data are shown as mean ± standard deviation (SD). *p<0.05 *vs.* age-matched WT mice (Mann Whitney test, performed per timepoint). Figure is adapted from Eijgenraam *et al*. with permission [2].

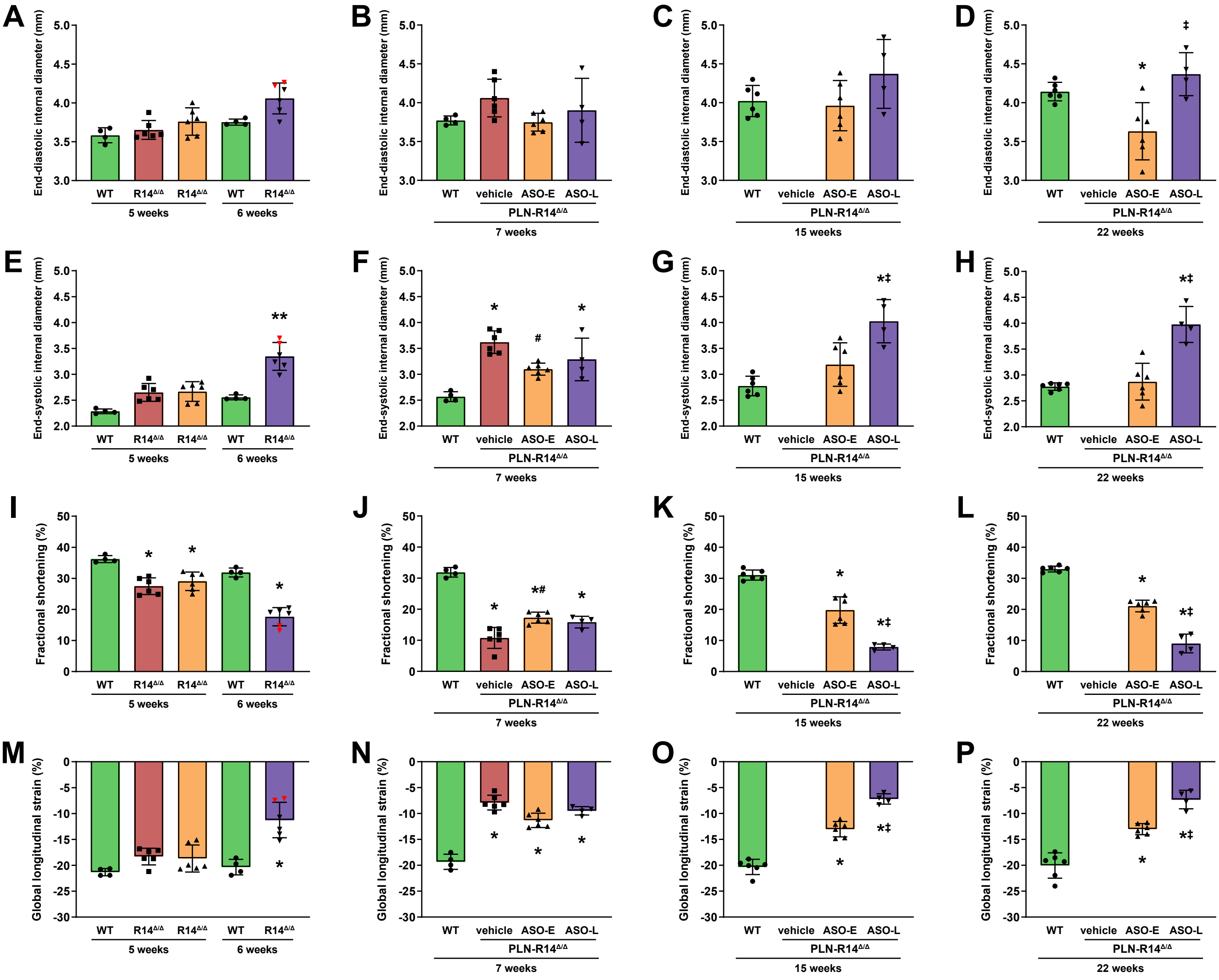

Figure S2. Echocardiographic parameters per time point of data shown in Figure 2. LV end-diastolic (**A-D**) and end-systolic (**E-H**) internal diameters, percentage fractional shortening (**I-L**) and percentage global longitudinal strain (**M-P**) at treatment initiation (5 or 6 weeks) (**A,E,I,M**) first (7 weeks) (**B,F,J,N**) and second (15 weeks) follow-up (**C,G,K,O**) or sacrifice (22 weeks) (**D,H,L,P**) in WT mice and PLN-R14$^{\Delta/\Delta}$ mice receiving vehicle or early or late PLN antisense oligonucleotide (ASO) injections (n=6 per group, except n=4 for 5-, 6- and 7-week-old WT mice and for 7-, 15- and 22-week-old ASO-late mice). Data of the two mice that died prematurely are marked with red symbols. Data are shown as mean ± SD. *p<0.05 vs. age-matched WT mice, $^{\#}$p<0.05 vs. age-matched PLN-R14$^{\Delta/\Delta}$ + vehicle, ‡p<0.05 vs. age-matched PLN-R14$^{\Delta/\Delta}$ + ASO-early (two-way ANOVA followed by Tukey's post-hoc test). Data of 5- to 7- week-old WT mice are derived from Eijgenraam *et al*. [2].

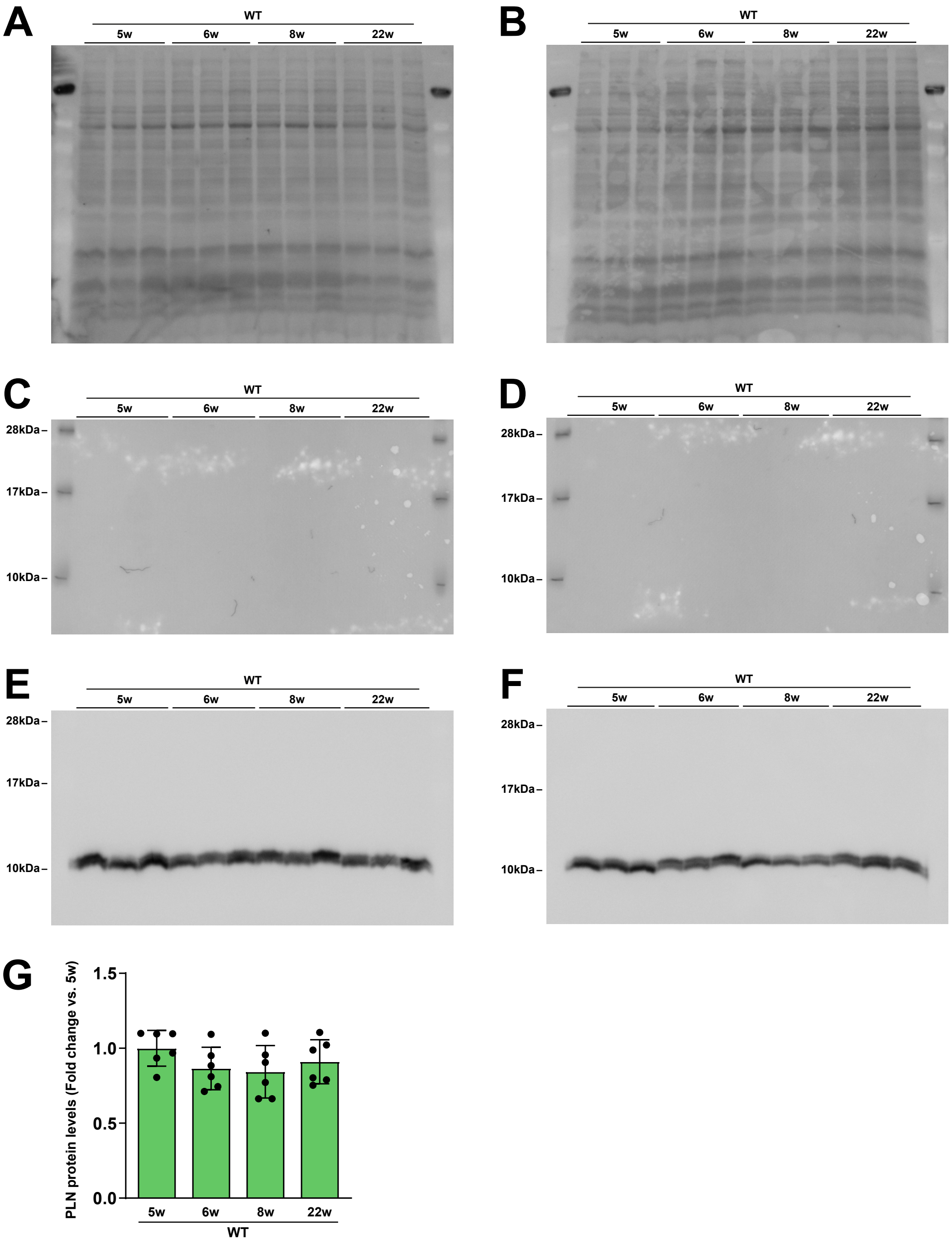

Figure S3. Phospholamban protein expression in 5- to 22-week-old WT mice. (**A-B**) Full blot images of total protein levels. (**C-D**) Full blot digital images after membranes were cut at 30 kDa, visualizing dye-stained molecular weight markers. (**E-F**) Full blot chemiluminescence images, revealing PLN protein levels. (**G**) Quantification of LV PLN protein expression normalized to total protein levels in 5-, 6-, 8- and 22-week-old WT mice, quantified as fold change compared to 5-week-old mice (n=6 per group). Data are shown as mean ± SD.

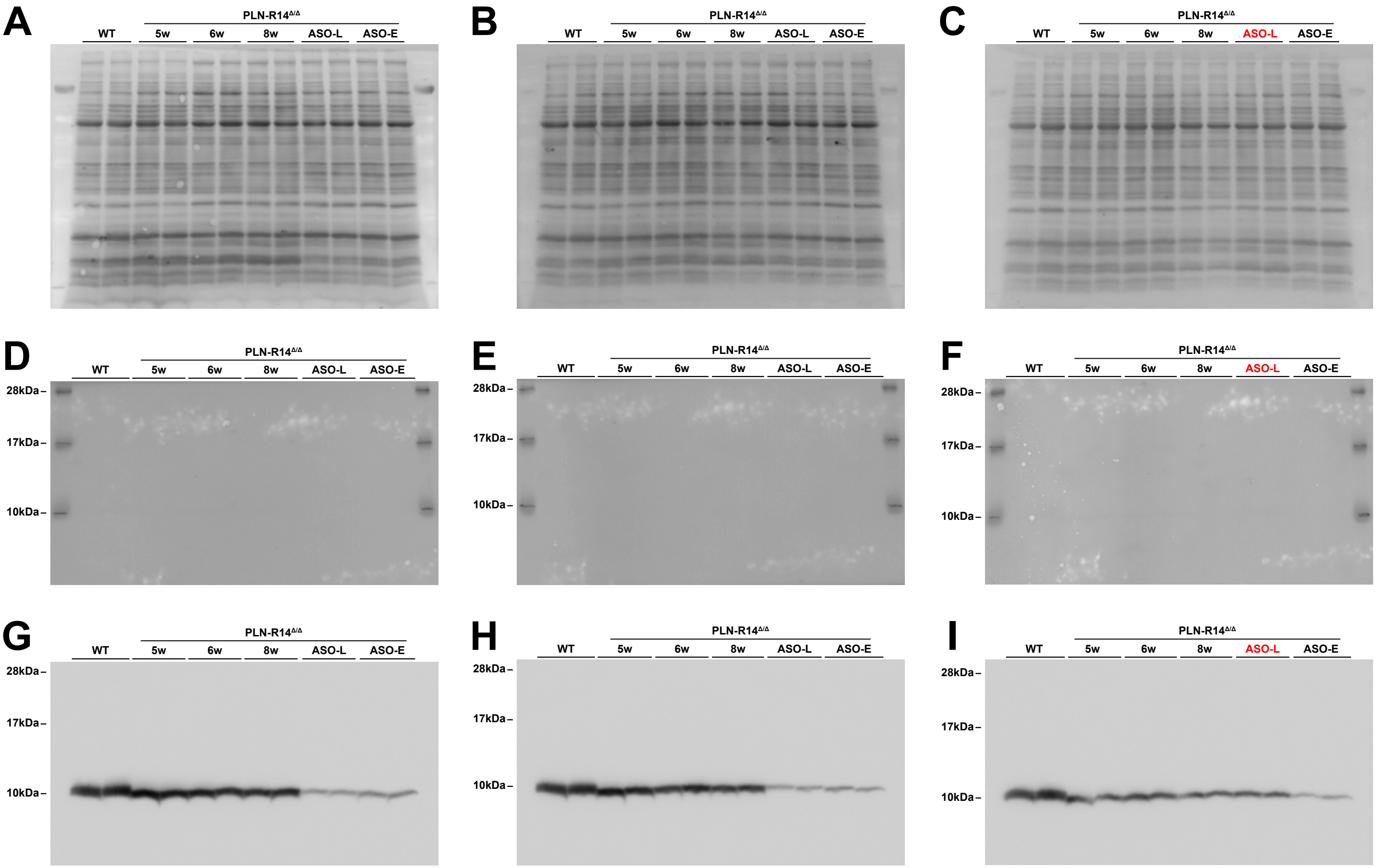

Figure S4. Full blot images of the PLN blots that are shown in Figure 3. (**A-C**) Full blot images of total protein levels. (**D-F**) Full blot digital images after membranes were cut at 30 kDa, visualizing dye-stained molecular weight markers. (**G-I**) Full blot chemiluminescence images, revealing PLN protein levels. Data of the two mice that died prematurely are marked with red text.

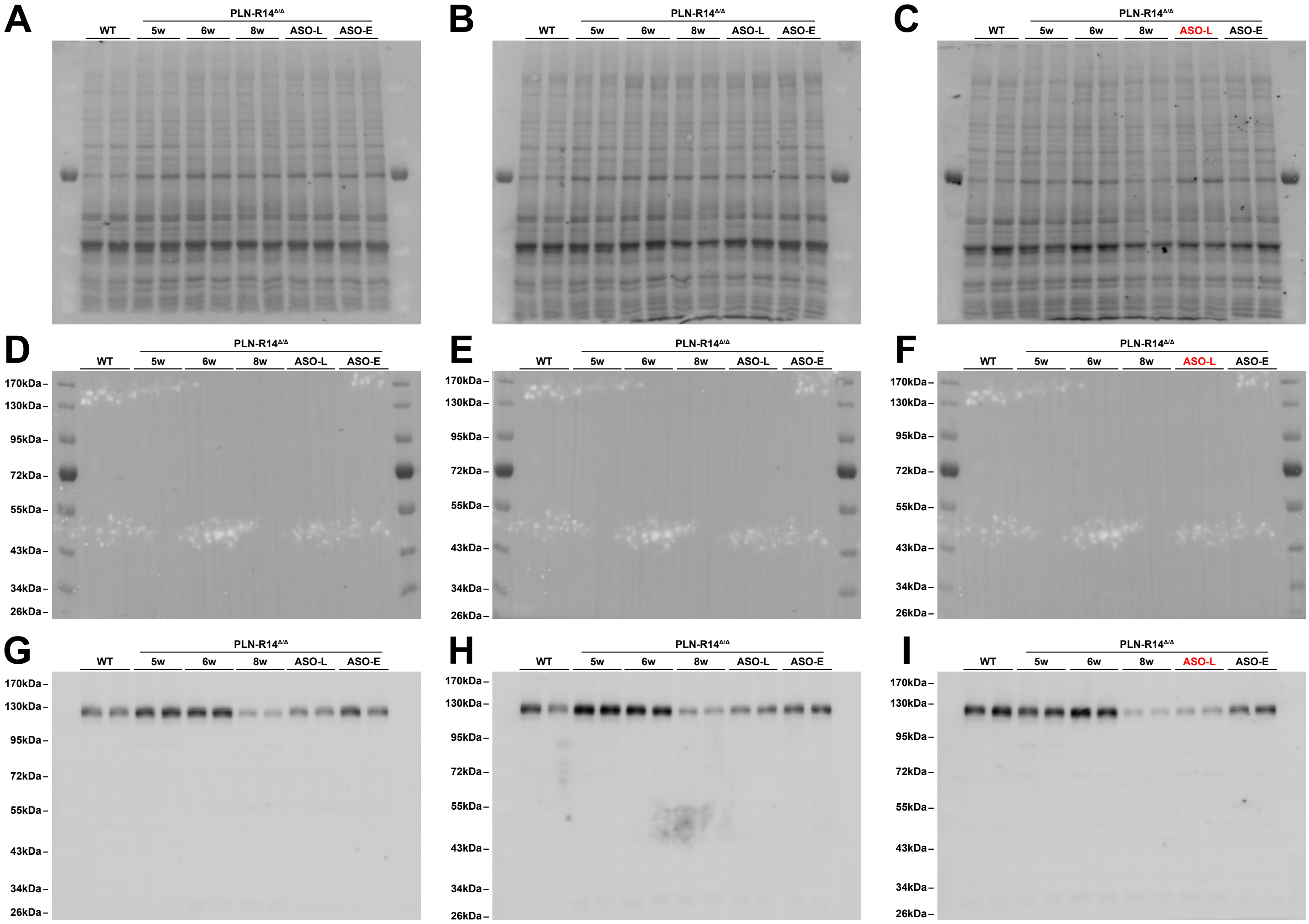

Figure S5. Full blot images of the SERCA2 blots that are shown in Figure 3. (**A-C**) Full blot images of total protein levels. (**D-F**) Full blot digital images, visualizing dye-stained molecular weight markers. (**G-I**) Full blot chemiluminescence images, revealing SERCA2 protein levels. Data of the two mice that died prematurely are marked with red text.

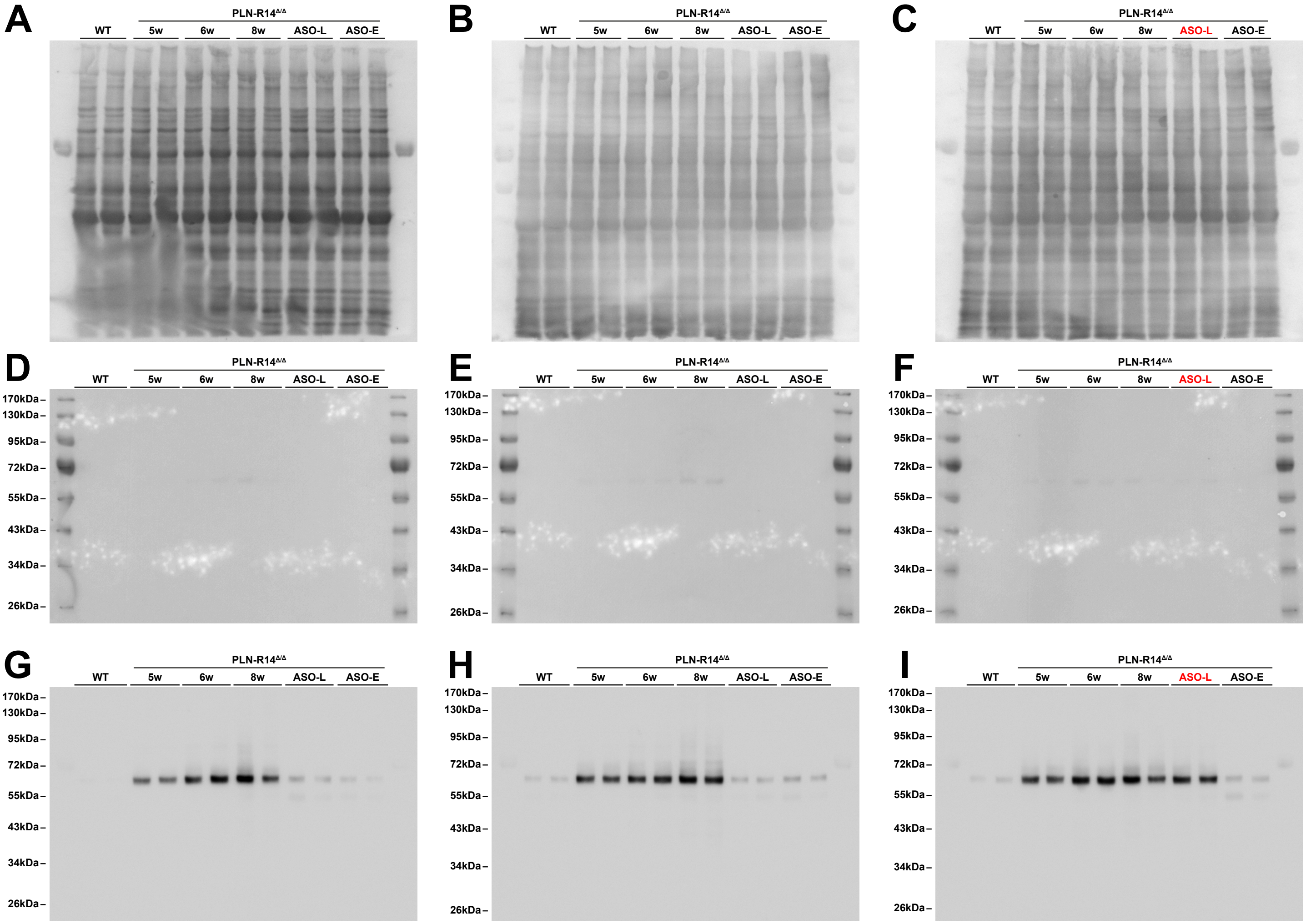

Figure S6. Full blot images of p62 blots that are shown in Figure 5. (**A-C**) Full blot images of total protein levels. (**D-F**) Full blot digital images, visualizing dye-stained molecular weight markers. (**G-I**) Full blot chemiluminescence images, revealing p62/SQSTM1 protein levels. Data of the two mice that died prematurely are marked with red text.

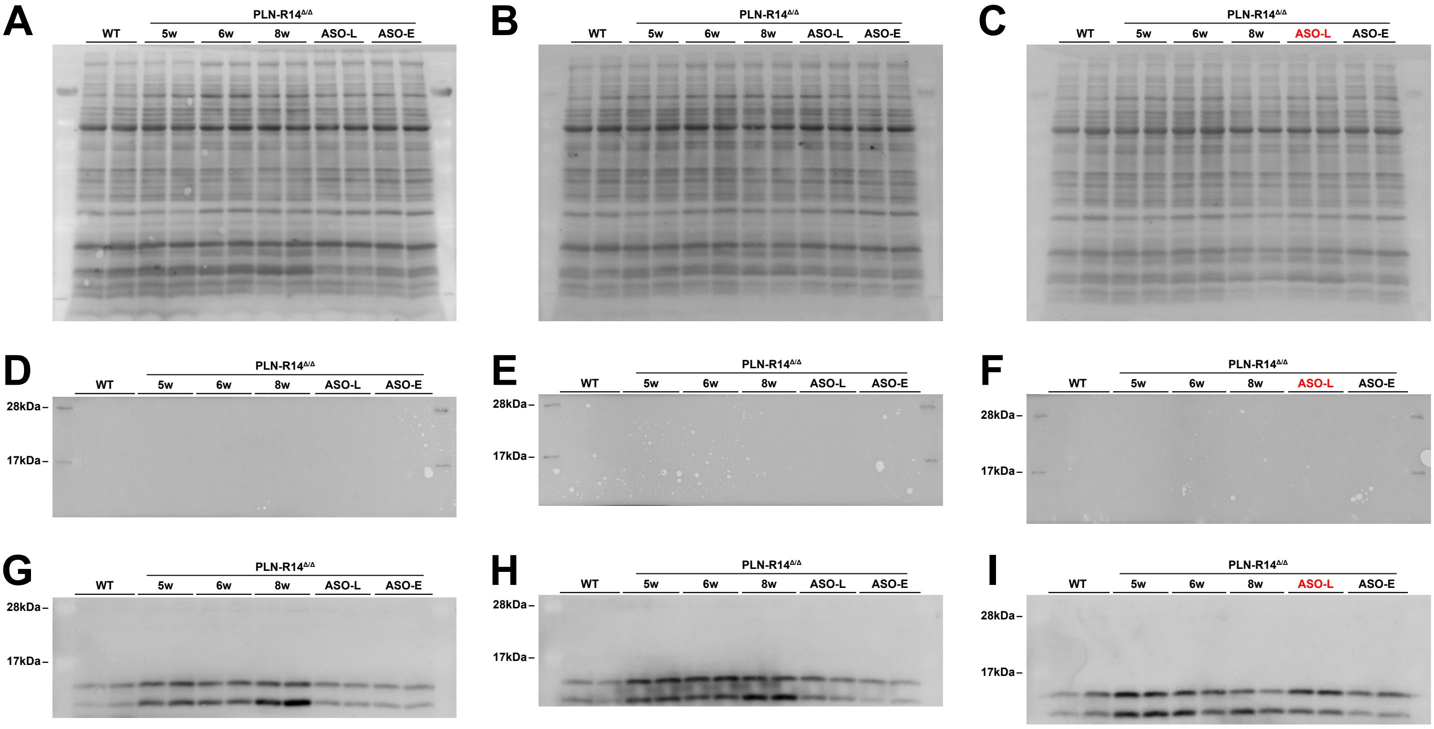

Figure S7. Full blot images of LC3 blots that are shown in Figure 5. (**A-C**) Full blot images of total protein levels (same blots were used as shown in **Figure S4**, but the blot was cut above the 10 kDa marker to remove the signal of anti-PLN antibodies, thereby circumventing the need for stripping of antibodies). (**D-F**) Full blot digital images, visualizing dye-stained molecular weight markers. (**G-I**) Full blot chemiluminescence images, revealing LC3 protein levels. Data of the two mice that died prematurely are marked with red text.

3. Supplementary tables

Table S1. List of primer sequences used in this study for qPCR.

Transcript	Name	Forward primer (5′-3′)	Reverse primer (5′-3′)	Product size
Col1a1	COL1A1	AGAGCATGACCGATGGATTC	CGCTGTTCTTGCAGTGATAG	138 bp
Nppa	ANP	GCTTCCAGGCCATATTGGAG	GGTGGTCTAGCAGGTTCTTG	86 bp
Pln	PLN	TGACGATCACCGAAGCCAAG	CAACAGGCAGCCAAATGTGAG	60/187 bp
Rplp0	36B4	AAGCGCGTCCTGGCATTGTC	GCAGCCGCAAATGCAGATGG	98 bp

Table S2. List of primary antibodies used in this study for Western blot.

Antigen	Supplier	Cat. no.	Host	Dilution	Block buffer
LC3A/B	Cell Signaling Technology, MA, USA	12741	rabbit	1:1,000	5% BSA-TBST
PLN	Cell Signaling Technology	14562	rabbit	1:1,000	5% BSA-TBST
p62/SQSTM1	Abcam, UK	ab56416	mouse	1:1,000	5% BSA-TBST
SERCA2a/b	Invitrogen	MA3-919	mouse	1:1,000	5% milk-TBST

Table S3. List of secondary antibodies used in this study for Western blot.

Antigen	Supplier	Cat. no.	Host	Dilution	Block buffer	Label
rabbit Ig's	Agilent Technologies	P044801-2	goat	1:2,000	5% milk-TBST	HRP
mouse Ig's	Agilent Technologies, CA, USA	P026002-2	rabbit	1:2,000	5% milk-TBST	HRP

4. Supplementary references

1. Eijgenraam, T.R.; Boukens, B.J.; Boogerd, C.J.; Schouten, E.M.; van de Kolk, C.W.A.; Stege, N.M.; te Rijdt, W.P.; Hoorntje, E.T.; van der Zwaag, P.A.; van Rooij, E.; et al. The Phospholamban p.(Arg14del) Pathogenic Variant Leads to Cardiomyopathy with Heart Failure and Is Unresponsive to Standard Heart Failure Therapy. *Scientific Reports* **2020**, *10*, 9819, doi:10.1038/s41598-020-66656-9.
2. Eijgenraam, T.R.; Boogerd, C.J.; Stege, N.M.; Oliveira Nunes Teixeira, V.; Dokter, M.M.; Schmidt, L.E.; Yin, X.; Theofilatos, K.; Mayr, M.; van der Meer, P.; et al. Protein Aggregation Is an Early Manifestation of Phospholamban p.(Arg14del)-Related Cardiomyopathy. *Circulation: Heart Failure* **2021**, *14*, e008532, doi:10.1161/CIRCHEARTFAILURE.121.008532.
3. Grote Beverborg, N.; Später, D.; Knoell, R.; Hidalgo, A.; Yeh, S.T.; Elbeck, Z.; Silljé, H.H.W.; Eijgenraam, T.R.; Siga, H.; Zurek, M.; et al. Phospholamban Antisense Oligonucleotides Improve Cardiac Function in Murine Cardiomyopathy. *Nature Communications* **2021**, *12*, 5180, doi:10.1038/s41467-021-25439-0.
4. te Rijdt, W.P.; van der Klooster, Z.J.; Hoorntje, E.T.; Jongbloed, J.D.H.; van der Zwaag, P.A.; Asselbergs, F.W.; Dooijes, D.; de Boer, R.A.; van Tintelen, J.P.; van den Berg, M.P.; et al. Phospholamban Immunostaining Is a Highly Sensitive and Specific Method for Diagnosing Phospholamban p.Arg14del Cardiomyopathy. *Cardiovascular Pathology* **2017**, *30*, 23–26, doi:10.1016/j.carpath.2017.05.004.
5. te Rijdt, W.P.; van Tintelen, J.P.; Vink, A.; van der Wal, A.C.; de Boer, R.A.; van den Berg, M.P.; Suurmeijer, A.J.H. Phospholamban p.Arg14del Cardiomyopathy Is Characterized by Phospholamban Aggregates, Aggresomes, and Autophagic Degradation. *Histopathology* **2016**, *69*, 542–550, doi:10.1111/his.12963.
